# Factors associated with DELAY in diagnosis among tuberculosis patients in Hohoe Municipality, Ghana

**DOI:** 10.1186/s12889-015-1922-z

**Published:** 2015-07-29

**Authors:** Eric Osei, Patricia Akweongo, Fred Binka

**Affiliations:** Department of Population and Behavioural Sciences, School of Public Health, University of Health and Allied Sciences, Hohoe, Ghana; Department of Epidemiology, School of Public Health, University of Ghana, Accra, Ghana; University of Health and Allied Sciences, Ho, Ghana

**Keywords:** Diagnosis, Patient delay, Tuberculosis, Patients, Hohoe-Ghana, Healthcare service

## Abstract

**Background:**

Any delay in diagnosis and consequently treatment of TB patients not only increases the infectivity of the disease in the community, but may also lead to more advance disease state, which may result in more complications and expose patients to higher risk of death. The aim of this study was to assess delays in diagnosing new TB patients and the factors associated with these delays in Hohoe Municipality of Ghana.

**Methods:**

A cross sectional study was carried out among 73 new TB Patients, 15 years or older, registered between 1st June, 2013 and 31st May, 2014 in Hohoe Municipality. Questionnaires were administered to patients to evaluate factors related to delay by patients in seeking care, delays at healthcare facilities, and total diagnostic delay. Logistic regression was used to determine the factors associated with patient delay (>30 days), healthcare services delay (>15 days), and total delay (>45 days).

**Results:**

The median total delay was 104 days (inter-quartile range (IQR):17–191). The median patient delay was 59 days (IQR: 5–123), and the median healthcare services delay was 45 days (IQR: 38–128). Not medically insured (AOR = 6.12; 95 % CI: 1.26–29.88; P < 0.025) and perceived stigma (AOR = 5.30; 95 % CI: 1.33–21.18; P < 0.018) were risk factors associated with prolonged patient delay. Multiple healthcare contact following signs and symptoms (AOR = 10.26; 95 %CI: 2.95–35.72; P < 0.0001) was the only risk factor associated with prolonged healthcare services delay.

**Conclusion:**

There is a considerable delay in TB case detection mainly due to patients delay in seeking healthcare. The factors associated with patients’ delay include lack of medical insurance, perceived stigma, and making multiple healthcare encounters. Health system strengthening towards decentralizing TB diagnosis and management, raising public awareness about the disease, training of healthcare providers, and collaborating with non-formal healthcare providers may reduce long delays in the management of TB.

## Background

Tuberculosis (TB) remains a major cause of morbidity and mortality and a significant global public health problem affecting about one-third of the world’s population despite the implementation of preventive and control measures over the years. An estimated 8.6 million new cases of TB was reported globally in 2012 with 1.3 million deaths [1].

World Health Organization (WHO) indicates that the African Region has the highest rates of TB cases and deaths per capita and accounts for 24 % of the world’s cases [2]. Among other factors, the HIV co-infection has intensified the TB epidemic. Sub-Saharan Africa accounts for approximately 80 % of the world’s TB ⁄HIV co-infection cases [3].

Even though Ghana is not among the 22 high burden TB countries in the world, yet the disease is considered an important public health challenge [4]. According to the WHO, Ghana is ranked 38th high burden TB country among 145 countries in the world and 19th in Africa and thus TB is considered an important public health problem in the country. The attempt to control TB in Ghana dates back to the early 1900s and national TB control programme was established in 1994 [5]. TB diagnosis and treatment are provided free of charge for all patients in Ghana. TB is diagnosed among patients who present themselves at out-patient-department of healthcare facilities in the country with cough for at least two weeks [6]. Sputum smear microscopy remains the most common diagnostic tool for diagnosing patients suspected of having pulmonary TB. Diagnosis of pulmonary TB (PTB) is confirmed by the presence of Acid-Fast Bacilli (AFB) in at least one of two sputum sample during microscopic examination. Smear negative pulmonary TB is confirmed by chest X-ray and/or clinicians’ judgement based on clinical presentation and epidemiological history. Patients who are suspected of having extra-pulmonary TB are also referred for X-ray and diagnosis made by an experience physician [6].

The key elements of TB control programmes are early diagnosis and prompt initiation of effective chemotherapy. Thus, any delay in diagnosis and consequently treatment of TB patients not only increases infectivity in the community but may also lead to more advance disease state which may result in more complication and expose them to higher risk of death [7].

A number of studies have identified delays in diagnosing TB in low and high prevalent countries, vary significantly from 45 days in Angola [8] to 91 days in Pakistan [9].

A study conducted in Kumasi in Ghana in 1995 estimated total diagnostic delay to be 120 days. However, the study involved only smear positive pulmonary TB cases [10]. We therefore conducted this study among all categories of TB patients in Hohoe Municipality of Ghana to assess TB diagnostic delays related to the patient or to the healthcare services and to identify factors associated with these delays.

## Methods

### Study setting

The study was done in Hohoe Municipality in the Volta Region of Ghana. The 2014 population of the municipality is estimated at 187,028, 84 % of whom are rural inhabitants and about 55 % live on subsistence farming. Trading is a common economic activity in Hohoe with closed to 25 % of residents involved in petty trading. People travel from all over the country to Hohoe for trading activities. Food insecurity and poverty pose a huge challenge to the population in the study area. The public health sector has 20 health facilities; 1 hospital, 15 health centre, 1 clinic, and 3 Community-based Health Planning Services (CHPS) [11]. TB diagnostic services currently are available only at the Municipal hospital located in Hohoe Township and not in lower health facilities. Patients from rural areas presenting to clinics with symptoms suggestive of TB are therefore referred to the hospital for diagnosis. The TB control programme is integrated into the public health system in the municipality. Patients have free access to TB and HIV diagnostic services in the Municipality even without being medically insured.

### Study design and population

**Fig. 1:**
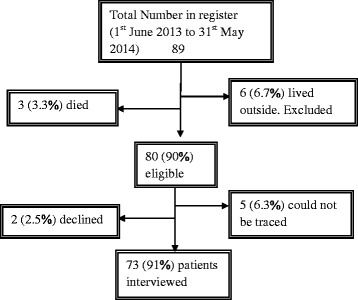
Patient recruitment processes

The study was a cross-sectional design based on patients’ records and interviews during home visits. Study participants included new TB patients, 15 years or older who were registered between 1st June, 2013 and 31st May, 2014. Patients who resided outside the study area as at the time of diagnosis and those with previous history of TB treatment where excluded from the study. Figure 1 below shows the patient the recruitment process.

### Instrument and data collection

The study was carried out using a pre-coded closed ended questionnaire. Data was collected based on i) socio-demographic variables: age, sex, occupation status, and education level; ii) health seeking behaviour: places of initial healthcare provider visited, number of healthcare services contact; iii) distance, time and two way cost of travel to reach TB diagnostic centre. Questions were also asked about the factors that might influence patients’ health-seeking behaviour, such as fear of social isolation, stigma, and knowledge of TB transmission and causes. A team of health personnel who had been working in TB control were trained to collect the data. Patients were asked to estimate the time in days from the onset of first TB symptoms to the first contact with health services (either public or private). Patients with three unsuccessful follow up attempts were considered untraceable. Routine data on sputum smear microscopy, date of diagnosis, HIV status, type of TB, age, sex and patients’ residence were extracted from the hospital TB register. For quality control, the study was piloted at non-study site prior to the commencement of the study for clarity and cultural acceptability of questions.

### Operational definitions

In this study we used the following operational definitions:

Patient delay was defined as the time interval between the onset of the first Tuberculosis (TB) symptoms and first contact with Public health services (health centres, hospitals or TB treatment centres) because of those symptoms.

Healthcare services delay was defined as the time interval between the first medical consultation to Public Health facility and the time taken to diagnose TB patient.

Total delay was defined as time interval between the first TB symptoms and TB diagnosis (which is equal to the sum of patient and Healthcare services delay).

New TB patient refers to a patient who has never had treatment for tuberculosis or who has taken anti-tuberculosis drugs for less than one month.

Pulmonary Tuberculosis (PTB) refers to a patient with tuberculosis disease involving the lung parenchyma.

Smear positive PTB refers to a patient with at least one sputum smear positive for acid fast bacilli (AFB), or one sputum smear positive for AFB plus radiographic abnormalities consistent with active pulmonary tuberculosis; or one sputum specimen positive for AFB plus culture specimen positive for *Mycobacterium tuberculosis*.

Smear-negative PTB refers to a patient with two negative sputum smears for AFB and radiological abnormality consistent with active TB or failure to respond to antibiotics treatment or one which health worker or clinician has diagnosed TB and decided to treat the patient with full course of anti TB drugs.

Extra pulmonary TB (EPTB) refers to patients with TB in any organ other than the lungs verified by histopathology.

Alternative care providers: These include traditional health providers, local injectors and drug retail outlets.

Stigma was constructed using social and psychological constructs of stigma (shame, moral value of blame, responsibility, and quilt) associated with Tuberculosis at both community and patient/individual levels.

Multiple Healthcare contacts was defined as making more than one visit to any Public Health facility before diagnosis.

Employment was defined as having income generating job.

### Data analysis

Data were double entered and cleaned using EpiData Version 3. Analysis was carried out using STATA statistical package Version 11 (Stata Corp, Collage Station). Frequency distributions and descriptive statistics such as mean/median and interquartile range (IQR) were calculated. Comparisons between groups were made using the Pearson chi-square test for qualitative/categorical variables (proportions), and the students’t-test for quantitative variables (means). Beyond descriptive statistics, associations between the dependent variables (patient delay, health service delay, and total diagnostic delay) and the independent variables were analyzed by calculating the Odds Ratios and 95 % confidence interval. First, the potential determinants were evaluated for their individual association with each type of delay in a univariable analysis. Secondly, multiple logistic regression analysis was performed to adjust for possible confounding effect of determinants associated with delays. All exposure variables that showed marginal association with outcome variables (p < 0.20) in the univariable analysis were included in the multivariable model.

The composite measure of patients’ knowledge was measured by the total number of correct answers to 7 items on knowledge of TB. These included knowledge about the causes of TB, curability, treatment duration, and existence of vaccine. The scale was then dichotomized into two categories (good or poor knowledge) using the median as the cut-off. Stigma was measured on a four-point Likert scale; 0 = highest, 3 = lowest degree of stigma). Variables for measuring stigma included feeling ashamed of having TB, having to hide TB diagnosis from others and having problems with family relations, work performance, marriage prospects, family responsibilities, infertility, or pregnancy. The index was then dichotomized into two categories (presence or absence of stigma) using the median value as the cut-off.

Distribution of delay data was checked for normality by visually inspecting the graphs created using delay data. Delays were skewed so median and interquartile range were reported together with means. Significance tests were two-sided and P-values of <0.05 was considered statistically significant. Prolong patient delay was defined as >30 days, whiles healthcare services delay was considered to be >15 days.

### Ethical issues

This study was approved by the Ghana Health Services Ethical Review Board. Each patient was informed about the objectives of the study and his/her right to decline participation. Written informed consent was obtained from all patients before interviews.

## Results

### Socio demographic characteristics of patients

Overall, 89 patients were registered in the Municipality during the period of 1st June, 2013 to 31st May, 2014. Of these, 80 (90 %) fulfilled the inclusion criteria and 73 (91 %) interviewed. All of the 73 patients were pulmonary TB cases of which, 21 (28.8 %) were smear positive and 52 (71.2 %) smear negative. Sputum culture was not done for all the smear negative patients since the service was not available during diagnosis. All the cases had their HIV sero-status checked, with 10 (13.7 %) being HIV positive. The 73 participants were between 15 and 88 years of age with mean age of 48.8 years (SD: ±17; median: 49; inter-quartile range (IQR): 25–73; 95 % CI: 44.9–52.8). Males and females were 48 (65.8 %) and 25 (34.2 %) respectively. Forty-five (61.6 %) came from rural with 41 (56.2 %) covering a distance of more than 5 km to the TB diagnostic centre. Sixty-five (89 %) patients had attended formal education of which, only12 (18.5 %) attained at least up to the secondary level. Forty-five (61.6 %) patients reported not having any income generation job (Table 1).

**Table 1:** Socio-demographic characteristics of new TB patients in Hohoe

Characteristics	Male		Female		All		Pearson Χ^2^
	n (%)		n (%)		N (%)		(p-value)
	48 (65.8)		25 (34.2)		73 (100)		
	No.	%	No.	%	No.	%	
Age (years)							1.98
							(0.577)
15 - 24	3	6.3	3	12.0	6	8.2	
25 - 34	7	14.6	4	16.0	11	15.1	
35-44	9	18.8	2	8.0	11	15.1	
>44	29	60.4	16	64.0	45	61.6	
Employment status							0.65
							(0.420)
Not employed	28	58.3	17	68.0	45	61.6	
Employed	20	41.7	8	32.0	28	38.4	
Highest Educational level							3.23
							(0.199)
Secondary+	8	16.7	4	16.0	12	16.4	
Primary/JHS*	37	77.1	16	64.0	53	72.6	
No education	3	6.3	5	20.0	8	11.0	
Residential status							0.51
							(0.474)
Urban	17	35.4	11	44.0	28	38.4	
Rural	31	64.6	14	56.0	45	61.6	
Medical Insurance							2.19
							(0.139)
Present	35	72.9	21	84.0	56	76.7	
Absent	13	27.1	4	16.0	17	23.3	
Sputum smear Status							1.13
							(0.288)
Negative	32	66.7	20	80.0	52	71.2	
positive	16	33.3	5	20.0	21	28.8	
HIV sero status							0.17
							(0.680)
Negative	42	87.5	21	84.0	63	86.3	
Positive	6	12.5	4	16.0	10	13.7	

### Health seeking trajectories

Nearly 14 % (10) of patients practiced self-medication following the onset of signs and symptoms, 29 (39.7 %) reported seeking healthcare first from alternative care providers.

Patients who first sought care from alternative care providers were similar to those who initially went to a public health provider with regards to age (p = 0.37), sex (p = 0.64), residence (p = 0.16), and HIV status (p = 0.17). Alternative care providers initially consulted were drug store/pharmacy (16, 21.9 %) and traditional healers (2, 2.7 %). Fourteen percent (10) practiced self-medication following the onset of signs and symptoms. Fifty-seven (78.1 %) had valid medical insurance card before seeking care. The majority (69, 94.5 %) of the patients presented with a combination of signs and symptoms with cough (71, 97.3 %) being the commonest complaint. Other signs and symptoms presented included fever (25, 34.2 %), chest pain (32, 43.8 %), weight loss (11, 15.1 %), haemoptysis (12, 16.4 %), and general fatigue (9, 12.3 %). Thirty patients (41.1 %) were diagnosed after one health care contact, 14 (19.2 %) after two visits, 15 (20.6 %) after three visits, and 14 (19.2 %) after four or more visits.

### Respondents’ knowledge and perceived stigma regarding tuberculosis

Before developing TB, 15 (21 %) (95 % CI: 12.0–31.6) of the patients had not heard of the disease. There was a significant difference between males and females regarding previous awareness of the disease; the proportion of males who had not previously heard of TB was significantly lower (5/48, 10.4 %) compared with 40 % (10/15) for females (p = 0.003). Patients who attained formal education to at least the primary level were more likely to have previous knowledge of TB compared to patients with no formal education (p < 0.0001).

Twenty-eight (38.3 %) of patients felt ashamed about developing Tuberculosis and more than one quarter had to hide the disease. Nearly 29 % (21) of patients said that Tuberculosis affects family relation, while 17 (23.3 %) said marital relation was affected by Tuberculosis.

### Pattern of delays

The time delays experienced by the patients are described in Table 2. Forty-four (60.3 %) (95 % CI: 48.1–71.5) patients had prolonged patient delay (i.e. >30 days). Prolonged healthcare services delay and total delay were observed among 52 (71.2 %; 95 % CI: 59.4–81.2) and 60 (82.2 %; 95 % CI: 71.5–90.2) patients respectively. The median patient delay was 59 days (IQR: 5–123 days). Patient delay was longer than 60 days in close to half (46.6 %) of the patients. The longest patient delay was 198 days and the minimum period of days in seeking TB care was 5 days following symptoms. Median patient delay among smear positive pulmonary TB patients (47 days) was lower than that of smear negative patients (59.5 days). The median patient delay among HIV sero-negative patients was 59 days whilst that of HIV positive patients was 57 days. HIV positive patients were 0.99 times less likely to delay seeking TB care compared to HIV negative patients, however, this difference did not reach statistical significant. (p = 0.985, 95 % CI: 0.25–3.85). The median healthcare services delay was 45 days (IQR: 38–128 days). Of the 73 patients, eight (11 %) were diagnosed on the same day they presented to a public healthcare facility, 17 (23.3 %; 95 % CI: 14.2–34.6) were diagnosed within 1 week of first contact with a public healthcare facility, 28 (38.4 %; 95 % CI: 27.2–50.5) within 30 days, and 28 (38.4 %; 95 % CI: 27.2–50.5) remained undiagnosed 60 days after initial visit to public healthcare facility. Median total delay was 104 days (IQR: 17–191 days). Total diagnostic delay was the same among smear positive patients and smear negative patients (104 days). A longer total diagnostic delay was noted for HIV positive patients compared to HIV negative patients (190 vs. 104 days),

**Table 2:** Distribution of various time delays (days) among 73 new TB patients in Hohoe, Ghana

Type of delay	Mean	SD	Median	IQR	Min	Max
Patient delay	55.3	40.0	59	5-123	5	198
Healthcare services delay	76.5	91.2	45	38-128	0	371
Total diagnostic delay	131.4	94.3	104	17-187	14	401

### Factors associated with delays

Being in employment (OR: 2.87; 95 % CI: 1.02–8.09; P < 0.046), not medically insured (OR: 4.04; 95 % CI: 1.05–15.65; P < 0.043), poor knowledge on TB (OR: 5.06; 95 % CI: 1.04–24.63: P < 0.045), and stigma regarding TB (OR: 4.0; 95 % CI: 1.29–12.40; P < 0.016) were associated with an extended patient delay on univariable analysis (Tables 3 and 4). On multivariable analysis, not have medical insurance (AOR: 6.12; 95 % CI: 1.26–29.88; P < 0.025) and perceived stigma (AOR: 5.30; 95 % CI: 1.33–21.18; p < 0.018) were associated with an extended patient delay (Table 5). Sputum smear negative patients were 58 % more likely to delay their first consultation with public healthcare services than patients with sputum smear positive; however, this was not statistically significant.

**Table 3:** Demographic and clinical factors associated with delays in Tuberculosis diagnosis

Variable	Patient delay			Healthcare services delay			Total delay		
	OR	95 % CI	*P*	OR	95 % CI	*P*	OR	95 % CI	*P*
Sex									
Male	1			1			1		
Female	0.98	0.37-2.64	0.972	1.44	0.48-4.33	0.517	1.93	0.48-7.77	0.355
Age (years)									
15–24	1		0.725^*^	1		0.534^*^	1		0.857^*^
25–34	1.33	0.15–11.50		2.25	0.23–22.14		0.90	0.64–12.58	
35–44	0.88	0.11–7.11		0.60	0.08–4.76		0.53	0.04–6.65	
>44	0.63	0.10–3.77		1.38	0.22–8.50		1.09	0.11–10.76	
Employment status									
Unemployed	1			1			1		
Employed	2.87	1.02–8.09	0.046	0.77	0.27–2.16	0.616	1.5	0.41–5.43	0.537
Highest Educational level									
Secondary+	1			1		0.913^*^	1		0.748^*^
Primary/JHS	0.70	0.19–2.63	0.603	1.23	0.33–4.84		1.63	0.37–7.23	
None	0.83	0.13–5.40	0.848	1.50	0.20–11.10		2.33	0.20–27.57	
Residential status									
Urban	1			1			1		
Rural	0.46	0.17–1.25	0.128	0.55	0.18–1.63	0.278	0.42	0.10–1.68	0.221
Medical Insurance									
present	1			1			1		
Absent	4.04	1.05–15–65	0.043	0.67	0.21–2.13	0.499	1.83	0.36–9.23	0.462
Sputum smear status									
Positive	1			1			1		
Negative	1.58	0.57–4.40	0.383	0.70	0.22–2.25	0.553	1.72	0.49–6.03	0.398
HIV status									
Negative	1			1			1		
Positive	0.99	0.25–3.85	0.985	1.73	0.33–8.91	0.514	0.85	0.16–4.54	0.846

*Likelihood ratio *p*-value

**Table 4:** Patient and health system-related factors associated with delays in Tuberculosis diagnosis in Hohoe, Ghana

Variable	Patient delay			Health services delay			Total delay		
	OR	95 % CI	P	OR	95 % CI	P	OR	95 % CI	P
Knowledge									
Good	1			1			1		
Poor	5.06	1.04–24.63	0.045	0.67	0.19–2.30	0.525	3.32	0.39–27.94	0.270
Stigmatization									
No stigma	1			1			1		
Stigma	4.0	1.29–12.40	0.016	0.45	0.16–1.27	0.130	1.93	0.48–7.77	0.355
Distance (km)									
>5	1		0.123^†^	1		0.298^†^	1		0.819^†^
5–8	0.45	0.16–1.29		0.43	0.14–1.34		0.68	0.18–2.53	
8+	1.75	0.40–7.69		0.93	0.20–4.35		1.02	0.17–6.01	
Time to reach the TB center									
<1/2 h	1		0.544^†^	1		0.565^†^	1		0.287^†^
½–1 h	1.7	0.58–4.94		1.82	0.55–6.07		3.03	0.59–15.73	
>1 h	1.7	0.43–6.65		0.96	0.24–3.83		0.83	0.18–3.80	
Cost of two way journey to Hospital (GHȼ)									
<5	1			1			1		
≥5	1.65	0.60–4.56	0.332	0.79	0.28–2.27	0.660	0.54	0.16–1.83	0.323
First provider visited									
PHF*	1			1			1		
Others	1.44	0.55–3.81	0.458	1.47	0.51–4.24	0.479	1.61	0.44–5.81	0.469
No. of Healthcare visits									
1	1			1			1		
2 or more	0.59	0.22–1.59	0.298	10.67	3.23–35.19	<0.0001	2.17	0.64–7.28	0.211

*PHF: Public Health Facility

*PHF: Public Health Facility

**Table 5:** Multivariable analysis of factors associated with delays in Tuberculosis diagnosis in Hohoe

	Patient delay			Healthcare services delay		
Variable	AOR^a^	95 % CI	P-value	AOR^b^	95 % CI	*P*-value
Employment status						
Unemployed	1					
Employed	2.61	0.73–9.29	0.139			
Residence						
Urban	1					
Rural	0.33	0.09–1.29	0.112			
Distance to hospital (km)						
<5	1					
5–8	0.57	0.15–2.16	0.407			
>8	5.78	0.86–38.90	0.071			
Medical insurance						
Present	1					
Absent	6.12	1.26–29.88	0.025			
Stigmatization						
No stigma	1			1		
Stigma	5.30	1.33–21.18	0.018	0.88	0.26–3.03	0.840
Knowledge						
Good	1					
poor	5.38	0.89–32.36	0.066			
No. of healthcare encounters						
1				1		
2 or more				10.26	2.95–35.72	<0.0001

^a^adjusted for employment, residence, distance, medical insurance, stigma, and knowledge

^b^adjusted for stigma and number of healthcare encounters

Multiple contacts to public healthcare provider prior to TB diagnosis (AOR: 10.26; 95 % CI: 2.95–35.72; P < 0.0001) was the overriding risk factor for prolonged healthcare services delay (Table 5). Females were 1.44 (95 % CI: 0.48–4.33) times more likely to have prolonged healthcare services delay than males. However, this association was not significant.

## Discussion

Key elements in any TB control programme are early diagnosis and prompt initiation of treatment. Analysis of factors associated with patient and healthcare services delay is an important step to identifying how to improve the quality of TB care and control. This study assessed delays and associated factors in the diagnosis of Tuberculosis in Hohoe Municipality, Ghana.

### Patient delay

Contrary to a previous report from Ethiopia [12] where majority (68.7 %) of their participants reported to a public medical provider within 30 days following the onset of symptoms. This study shows that more than 60 % of patients delayed their first visit to medical provider following symptoms for more than 30 days. Eleven percent of patients consult a healthcare provider within two weeks in this study, compared to 70 % reported by Kiwuwa and colleagues in Kampala Uganda [13]. Two weeks is generally the timeline recommended for a patient with cough to visit a health facility [14]. This long patient delay can reflect poor patients’ knowledge and awareness of TB symptoms and the need for prompt consultation with healthcare services for diagnosis and treatment. The proportion of patients (60.3 %) who delay seeking care for > 30 days is higher in this study than what was found in previous studies in Tanzania and Uganda [15, 17]. However, this study reports a lower proportion of patient delay than what was reported in Nigeria [16], where patient delay was observed in more than 80 % of patients. In a similar study done in Kumasi, Ghana in 1995 to determine the factors associated with diagnosis delay among pulmonary TB patients, a low proportion of patient delay (46 %) was reported [10]. Also, the median patient delay of 47 days among sputum smear positive patients in this study is longer than what Lawn and colleagues found in their Kumasi study. These differences in patient delay between the two studies could be due to study setting as more urban dwellers living close to health facilities may have shorter patient delay than rural dwellers. Sreeramareddy et al. in their systematic review [17], found that among low and middle income countries, patient delay varied from 4.9 days in Gambia to 162 days in Tanzania. It is likely that most of these studies may have underestimated patient delays since patients often do not clearly recognize symptoms at the onset of disease.

Patient delay has been reported to be more common among females than males [18]. This study however, finds an insignificant difference between the sexes and increased patient delay, a finding consistent with those reported in Tanzania [15], Nigeria [16], Ethiopia [19], Malaysia [20], and Norway [21], where sex was not found to be a significant predictor of prolong patient delay. In contrast to the Kumasi study [10], males in this study postponed care-seeking longer than females. In a study in India, males delay longer because of fear of cost of diagnosis and treatment [22]. Also, men were more likely to neglect symptoms longer until they are serious before seeking care. Fazlul and colleagues attributed similar differences in case detection to women’s limited decision-making power and failure of health systems to provide accessible and acceptable health care [23].

This current study shows that patients in employment have 2.9-fold greater median patient delay than the unemployed patients in the univariable analysis. A possible explanation for this variance can be that those employed are busy at their work and are not able to attend a clinic with their symptom.

In this study, patients who first sought care from alternative care providers following symptoms have an increased risk of patient delay, though not statistically significant. One can speculate that this group of patients did not perceive themselves to be at risk of TB and did not take their symptoms seriously enough to seek formal medical care.

Stigma plays an important role in determining the health-seeking behaviour of suspected TB patients [9]. Despite decades of public health efforts, stigma continue to impede progress in diagnosis and treatment of TB [24]. This study, like other studies [25-27] finds that stigma was independently associated with prolonged patient delay. Nonetheless, most of the patients are willing to reveal their illness to others in this study, this is similar to what was reported previously in Ethiopia where 88 % of study participants were willing to disclose their TB status to everyone [19]. Several studies have shown why and how TB has been highly stigmatized throughout history. Most authors recognize the perceived infectiousness of TB as a major cause of stigmatization [26-28]. Whilst the stigma of TB as “a disease of the poor” persists, more recently, HIV/AIDS stigma affects TB patients, particularly in communities where HIV/AIDS is prevalent as shown in studies in Ethiopia [27]. Dordor and Kelly [28] recognised that fear of stigma can result in infected individuals hiding their disease from their families and others

As found in China [29] and France [30], lack of medical insurance was surprisingly found to predict extended patient delay in this study. Though not explored in this study, lack of medical insurance may be related to poverty. Poverty and lack of insurance may discourage people from seeking prompt healthcare. In this view, Ghana National TB programme as in many TB endemic countries had implemented free TB diagnosis and treatment policy to reduce financial burden of patients and hence improve access to TB diagnosis and treatment. In addition to this free policy, the NTP had implemented patients’ “enablers” package to take care of other expenses such as transportation costs, medical consultation fee, cost of folders, chest X-ray, and food supplements for patients who cannot afford.

Factors such as age, educational level, knowledge, which have been identified to be significantly associated with patient delay [13, 19, 29,] could not be established in this study.

This study also failed to find an association between access to healthcare and prolonged patient delay. This may be due to the limited sample size obtained for the study.

This study did not find an increase in patient delay among rural dwellers compared to urban patients, despite the fact that these patients are likely to have poor access to healthcare and that they were found to be less educated compared to patients from urban settings. Yimer et al. [12] however, found in their recent study among 201 patients attending a referral hospital in Northwest Ethiopia that rural dwellers were 3.4 times more likely to have increased patient delay than urban dwellers, similar to previous report [31], suggesting that rural patients in this study setting has better access to healthcare than rural dwellers in Ethiopia.

### Healthcare services delay

In this study, the time period from patients’ first contact with public health services to diagnosis (45 days) is unacceptably long and contributes significantly to total diagnostic delay. This finding compares favourably with reports from other countries such as Ethiopia [32], Uganda [13], and Pakistan [9] that reported median healthcare system delay ranging between 61 days and 87 days, however, longer than report Nigeria ([16]. The differences can perhaps be due to the variation in the definition of healthcare system delay. Some studies defined it to include the period between TB diagnosis and initiation of treatment. In contrast to the patient delay, the median healthcare services delay in this study is shorter than the previous report from Ghana [10]. Lawn’s study was just a year after establishing a new TB control programme in the country and since then the National Tuberculosis Control Programme has been implementing activities towards improving quality of TB care, which include extensive training of health personnel, decentralization of diagnosis and treatment services, community TB-DOTS, and provision of “enabler” package to health providers and TB patients as well, aiming at providing a quality integrated TB services to people at all levels by means of standardised diagnosis, treatment and community-based care and support. These measures are expected to reduce diagnostic delays. This could explain the shorter median healthcare services delay in the current study. This study dismisses the view held by healthcare providers that the late presentation of patients is mainly “the patients’ doing”. On the contrary, in approximately half of the patients, the healthcare services delay exceeded the patient delay. This reflects the inefficiencies on the part of healthcare services in the diagnosis of Tuberculosis. Proportion of healthcare services delay in this study is higher among urban dwellers than rural patients (78.6 vs. 66.7 %), however, rural dwellers had longer healthcare services delay compared to patients from urban settings (median: 54 vs. 45 days). This is perhaps due to the fact that urban health providers are able to suspect and diagnose TB cases faster than their rural counterparts. The municipal hospital is the only health facility that provide diagnostic and treatment services for TB patients at the time of the study thus patients suspected in the rural facilities must be referred to the hospital for diagnosis. Some of these patients might have delayed at home after referral explaining the prolong time duration between patients first encounter with healthcare services and diagnosis.

In line with a previous study in Kampala, Uganda [33], this study shows that a significant proportion of TB patients used alternative healthcare as the first choice of care in Hohoe Municipality, with nearly 40 % initially presenting to pharmacy or drug shops, traditional healers, and private clinic. On the contrary, Pronyk et al. [34] reported that 75 % of patients first visited public health system. This may reflect lack of awareness of free TB diagnosis and treatment services as more than half of patients hoped to receive cheaper services at those places.

This study demonstrates that “multiple healthcare contacts” is the overriding predictor to healthcare services delay. Similar finding was reported in Afghanistan, where making more than one visit to health care providers strongly predicts increased health system delay [25]. This relationship may be associated with poor clinical suspicions of signs and symptoms by healthcare providers especially from primary healthcare facilities and failure to request for proper investigations or refer patients to TB centre for further investigations, which led to patients making multiple contacts because of persistent symptoms. Some authors attribute this delay to provider failure to correctly diagnose TB patient [10,13]. Wrishmeen and colleagues [25] speculate that multiple health care provider visits influence health system delay when patients receive inappropriate antibiotic that can modify the clinical picture which may cause patients in believing that they will be cured; and in the long run, may choose alternative treatment. Needham et al. [35] also indicated that centralized public services and a lack of integration between public and private providers prolonged health system delays.

Contrary to the previous report from Ghana [10], where doctor delay was 3.9 times greater for rural dwellers compared to patients from urban settings, this study found no increase in healthcare services delay among rural patients compared to urban dwellers. This suggest that both rural and urban healthcare providers contribute equally to healthcare services delay in Hohoe Municipality despite the fact that rural dwellers are likely to have poorer access to TB diagnosis services as the only diagnostic facility is located in Hohoe township.

### Total delay

The total median delay is 104 days, similar to what was found in Brazil [36] during which the patient was transmitting the infection to the close contacts in the community. Approximately 80 % of patients have total diagnostic delay exceeding 2 months, more than what was reported in Ethiopia (47.8 %) [12]. This has a great public health implication since increased transmission in communities may imply that patients may have waited longer in the communities before diagnosis and treatment. Long total delays have also been found in industrialized countries like the United Kingdom (78 days) [37] and United States (89 days) [38]. The median total delay of 104 days (3.5 months) is shorter than 4 months reported in the Kumasi study. In line with studies in Nigeria [16] and Nepal [39], patient delay is a major contributor to the total diagnostic delay in this study, contributing approximately three-fifth of the total delay, finding contrary to previous study in Kumasi where doctor delay was the main contributor to total delay [10]. The proportion of patients with total delay was higher among urban dwellers (89.3 %) than rural patients (77.8 %). Moreover, urban dwellers were more likely to have longer total delay than rural dwellers, which may imply that patients from urban settings delay longer before diagnosis to avoid disrupting perhaps their economic activities. Similar to a report from Brazil [36], more patients with AFB negative had longer total delay than AFB positive patients.

The most obvious limitation of the study is its small sample size. Therefore firm conclusions about the relationships among variables cannot be drawn. Thus interpretation of the results must be done with caution. Research studies with much larger sample size would therefore be required to ensure appropriate generalization of the findings of the study. Secondly, since only self-report measures were used to estimate delays, response consistency effects may have biased the observed estimates. However, measures were taken to minimize this limitation. For example interviewers used national and local events to estimate date of onset of symptoms. Secondly, the date of first consultation with healthcare provider was self-reported and could not be validated in this study thus leading to potential differential misclassification as a result of recall bias.

## Conclusions

There is considerably long delays in diagnosing TB patients which is contributed by patients’ failure to seek prompt and appropriate healthcare following the development of signs and symptoms (patient delay) and healthcare providers’ inability to diagnosis promptly suspected TB patients after contact with the health system (healthcare services delay). Patients without medical insurance prior to TB diagnosis and those with perception of stigma are more likely to experience longer patient delay, whiles those who made repeated visits to public healthcare facilities is the overriding factor that predict increased healthcare services delay.

Raising public awareness about the signs and symptoms of the disease and availability of free services, regular training and re-training of healthcare providers, and collaborating with non-formal healthcare providers could reduce long delays in the management of TB. Decentralization of TB services to the peripheries is highly recommended.
